# You “can’t get away from the social”: Individual, relational, and contextual influences on mental health and substance use-related help-seeking among people in contact with the criminal justice system

**DOI:** 10.1371/journal.pone.0354996

**Published:** 2026-07-30

**Authors:** Catriona Connell, Morgan Torrance, Jessica Greenhalgh, Dave Griffiths

**Affiliations:** 1 Salvation Army Centre for Addiction Services and Research, University of Stirling, Stirling, Scotland; 2 University of Stirling, Stirling, Scotland; University of Alcala Faculty of Medicine and Health Sciences: Universidad de Alcala Facultad de Medicina y Ciencias de la Salud, SPAIN

## Abstract

**Rationale:**

People in contact with the criminal justice system outside institutional settings have high levels of mental health and substance use problems, contributing to adverse outcomes. Help-seeking behaviour is an important component of optimising access to effective treatment and support that is under-explored in this population. Empirical investigation into the influences on help-seeking for stigmatised health conditions is needed to inform efforts to facilitate help-seeking.

**Aim:**

This study established the individual, relational, and contextual influences on help-seeking for mental health and substance use problems among people in contact with the criminal justice system living in the community, and examined the applicability of existing models and frameworks for understanding help-seeking behaviour in this population.

**Methods:**

We applied framework analysis to qualitative data from a mixed-methods social network analysis study. Our framework drew on help-seeking behaviour and healthcare access models to determine initial categories for help-seeking influences (desire, ability, and context).

**Results:**

Help-seeking was confirmed to result from an interaction between ‘desire to seek help’, ‘ability to seek help’, and ‘help-seeking context’. We show that help-seeking is not an individual behaviour, but strongly affected by relational influences that operate between individuals, across social networks, and via cultural norms. High levels of criminal justice involvement, and mental health and substance use problems in the social networks of people in contact with the criminal justice system shape these influences.

**Conclusions and implications:**

Optimising help-seeking for mental health and substance use problems among people in contact with the criminal justice system living in the community requires critical engagement with, and application of, existing models to ensure sufficient appreciation of relational and contextual influences. Research is needed to develop and test multi-level policy/practice interventions that go beyond individual factors.

## Introduction

Many people in contact with the criminal justice system serve all or part of their sentence in the community [1,2]. Mental health and substance use (alcohol and drugs) problems are prevalent and frequently co-morbid in this population [3–7]. Prevalence estimates vary [3] but typically report over 40% meeting diagnostic criteria for a substance use disorder [5], more than 10% for a major depressive episode or psychotic disorder [5], and 75% for any personality disorder [6]. Mortality rates are higher compared to the general population [8], with a substantial number of avoidable deaths attributed to substance use or self-harm [9]. It is vital to enable people in contact with the criminal justice system to access adequate, timely support in the community to improve outcomes.

Help-seeking is a central component of general and mental healthcare access [10–12]. Models of healthcare access acknowledge individual, social, relational, and structural factors. However, there is little explanation on how these factors influence *help-seeking* specifically, nor how help-seeking may differ for highly stigmatised conditions, including mental health and substance use problems [13]. Help-seeking behaviour is shaped by individual attitudes, appraisal of social norms, and sense of control over behaviour [14]. These variables (attitudes, norms, ability) are associated with mental health help-seeking intentions [15] and have been used to inform interventions to increase mental health help-seeking [16,17]. Results of general population surveys on help-seeking for mental health and substance use problems demonstrate associations between help-seeking behaviours and gender; perceptions of stigma; and knowledge, attitudes and beliefs towards mental illness/substance use and treatment [18–22]. Whilst large general population surveys give important insights into associations between individual factors and help-seeking, they are limited in their ability to explore social/relational influences (e.g., shared beliefs between network members), surrounding cultures (community suspicion of state providers), or the specific experiences of people in contact with the criminal justice system.

In health research [e.g., 23,24] and other fields, [e.g., 25,26] researchers applying social network theory and analysis have shown that social networks in social contexts influence behaviours via various mechanisms. Selectivity describes how people form networks around themselves made up of people with shared characteristics, experiences, values, or behaviour that reinforce their own beliefs, attitudes and behavioural choices [27]. Alternatively, people may adopt the attitudinal and behavioural norms of the network already surrounding them, described as contagion [28]. Appreciating how networks influence behaviour is important for interventions that go beyond an individual, to consider the wider context which may facilitate or inhibit intervention effects. However, research to understand the relational influences on help-seeking for stigmatised conditions among marginalised populations is limited and the distinct experience of people involved in criminal justice system is minimally explored [29].

Men in prison describe a reluctance to seek help for mental distress because of their perceptions of stigma, fear of showing vulnerability that may place them at risk, a desire to project strength and masculine ideals, mistrust of services, and negatives beliefs about the outcomes of seeking help [30]. On release from prison, competing financial and accommodation needs can contribute to individuals deprioritising help-seeking for mental health and substance use [31]. Scrutiny can cause stress and disrupt help-seeking activities [32,33]. Further, people in contact with the criminal justice system in the community disproportionately live in social contexts affected by socioeconomic deprivation [34,35] where mental health and substance use problems are more likely to be present in their social networks, and services may be limited or oversubscribed [36,37].

There is very limited literature exploring help-seeking among people in contact with the criminal justice system beyond prison. Qualitative investigation is required to understand the individual, relational, and contextual influences on help-seeking among people in contact with the criminal justice system living in the community. This can inform judgements about the applicability of existing help-seeking/healthcare access models, and inform design of optimally effective individual and community level interventions. We aimed to understand the influences on help-seeking for mental health and substance use among people in contact with the criminal justice system in community settings, and to summarise these in an accessible framework that could be used to design or deliver interventions to increase help-seeking and access to care.

## Methods

We report in-depth qualitative findings from a mixed-methods social network analysis study that explored how social networks influence help-seeking for mental health and substance use among people in contact with the criminal justice system in the community [38,39]. We collected qualitative data via interviews and applied framework analysis. Reporting is informed by COREQ [40].

### Inclusion criteria and sampling

Participants were adults (18 + years) who had been in contact with the criminal justice system, including courts, prisons, or community justice services. We limited this to contact in the last six months, to access contemporary experiences. Participants were included from two contrasting local authority (LA) areas in Scotland, to assist in drawing out context-specific influences. LA1 is largely rural and relatively affluent, with a local economy traditionally supported by agriculture and fishing. LA2 is geographically small, urban, and impacted by high levels of socioeconomic deprivation and declining heavy industry. Participants were excluded if taking part may negatively impact their health, sentence completion, or researcher safety, as determined by the recruiting organisation/staff member based on their knowledge of the person and their current presentation. Participants gave informed consent and were excluded if their mental state (including due to intoxication) prevented them from understanding the participant information at the time of interview. This was judged by organisational staff and by researchers taking informed consent.

We had capacity to interview 60 people. We adopted purposive and snowball sampling, aiming to over-recruit women (~5–10% of people in contact with the criminal justice system) and include participants with a range of ages and from different locales within each LA. We sought to balance sample size, demographics, sufficient data richness, and burden on supporting services. Sampling ceased based on a team judgement that we had more than sufficient qualitative data, and the benefit of further interviews was outweighed by the resourcing, logistical demands, and the burden on supporting services. This decision was taken separately in each LA.

### Recruitment

We worked with justice social work (who perform community supervision/probation functions in Scotland) and third/voluntary sector services to recruit. We met with service providers in advance to explain the research, what was involved, and how they could support recruitment and interviews. Service providers informed their service users via word-of-mouth and flyers. When someone agreed to participate, a researcher attended a location that the person was familiar with to provide study details and, if appropriate, obtain consent.

### Data collection

Three researchers (CC/MT/JG) conducted audio-recorded semi-structured interviews facilitated by social network data collection software, Network Canvas [41]. The interview was piloted with a Lived Experience Advisory Panel (LEAP; see below) and amended accordingly before use with participants. After collecting demographic details, participants were asked about experiences of mental ill-health, substance use, and seeking and accessing help. We asked about their perceptions of the helpfulness of different sources of support, and of mental health and substance use-related stigma. We asked about people in their social network, including the participant’s perceptions of each person’s attitude to help-seeking and their experience of mental health, substance use, and criminal justice problems. All data were self-reported. We collaboratively created a network diagram and explored the participant’s experience in their local context. Some questions were designed for quantitative data collection; however, our qualitative analysis includes the surrounding rich participant reflections and comments. The full interview protocol is available [39]. Interviews took place from 1^st^ November 2023–31^st^ July 2024 and were concluded when we had a sufficient sample size for the wider mixed-methods study, and sufficient rich data for qualitative analysis.

Interviews were typically conducted at the premises of the service provider who had facilitated recruitment, and thus were familiar to participants. One participant, recruited via snowballing, was interviewed at LA offices. Data were collected in a single interview to reduce participant burden. Interviews were transcribed verbatim and pseudo-anonymised prior to analysis. Interviewers retained notes and reflections of pertinent issues.

### Data analysis

**Fig 1:**
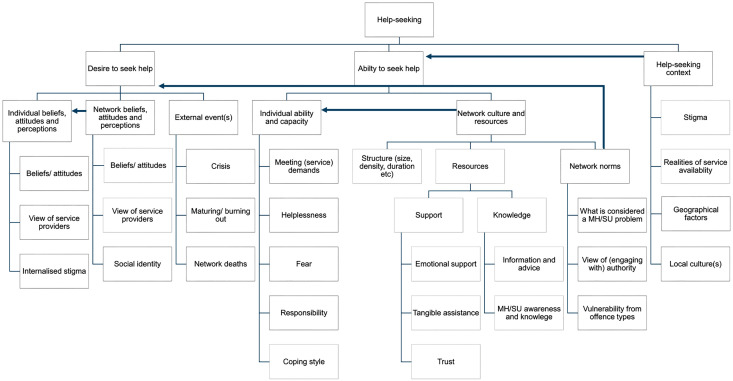
Framework of factors influencing help-seeking for mental health and substance use. (A) Coding framework showing themes and subthemes, representing influences on help-seeking and where these interrelate.

We adopted framework analysis combining deductive and inductive approaches, assisted by Nvivo software [42]. Our initial framework synthesised common help-seeking elements from conceptual models of mental healthcare pathways [11,12,43] and general healthcare access [10] to form initial categories (themes): desire to seek help, ability to seek help, and help-seeking context. Researchers deliberately attended to relational processes and social context, in addition to individual factors. Three researchers (CC/JG/MT) independently coded three transcripts to the initial framework, inductively creating sub-themes within each main category (desire, ability, context). We (CC/JG/MT) met to discuss utility and consistency, which was high at the main category level. A key area of discussion was how to represent the highly inter-related relational and individual elements of help-seeking behaviour. We considered an additional category/theme for relational factors but ultimately decided to represent relational elements as a sub-theme within each of desire and ability, highlighting its integral presence rather than something that can be separated. After refining and adding inductively identified sub-themes into the framework, and discussing and agreeing the optimal structure, two researchers (JG/MT) independently coded two further transcripts. Consistency remained high, including at sub-theme level, and all transcripts were then independently double coded to the framework. A third researcher (CC) checked coding and created a matrix, with cases as rows and themes/sub-themes as columns. Coded segments were pulled into the relevant cells and summarised, before synthesising the main elements of each theme/sub-theme, and final review by all researchers. Overlapping sub-themes were then combined with input from all analysing researchers (CC/JG/MT) to produce the final framework (See Fig 1). We maintained a record of each iteration of the framework.

### Patient and public involvement

We formed a Lived Experience Advisory Panel (LEAP) of people with experience of the criminal justice system and mental health or substance use-related help-seeking. The LEAP was coordinated by a Research Assistant with relevant lived experience and met throughout the wider study. The LEAP informed the interview design and approach, advised on software suitability, supported reflection on the findings and implications, and co-developed a comic as an accessible output [44]. Following feedback, we reduced the interview length and altered some language. We held regular discussions with service providers in the recruitment areas, and area representatives joined a study advisory group to assist with recruitment, interview scheduling, inform of practice changes/realities, and consider the implications.

### Research team and reflexivity

CC, PhD, is a senior researcher. MT and JG are Research Assistants, both MSc. All are white Scottish women under 40, familiar working with underserved populations affected by multiple deprivation in the UK. DG, PhD is a white male senior academic with expertise in social network analysis. Some members of the research team have personal/family and professional experience of the criminal justice system, mental ill-health, and substance use problems in addition to academic knowledge, which is not allocated to team members for privacy. CC designed the protocol with input from DG, provided training to MT and JG, and met weekly with MT and JG to provide support with challenging interview content/experiences, analysis and reflections, and to interrogate where emotion and assumptions may impact interpretations. The team (CC/JG/MT/DG) met regularly to discuss progress and analytical insights. Participants and the research team had no prior relationship. Participants were aware of the team’s employer, study funders, and approvers.

### Ethics

We obtained ethical approval from the General University Ethics Panel, University of Stirling (13947), and additional approvals from Community Justice Scotland and The Salvation Army. Participants gave verbal informed consent to participate, to be quoted verbatim, and for the pseudonymised data to be used in future research. The participant or researcher documented this by checking relevant boxes within the computer-mediated interview software.

## Findings

We interviewed 50 people: 23 in LA1 and 27 in LA2. An additional two wished to participate but did not have capacity to consent or did not meet inclusion criteria. No participants declined to participate or dropped out after agreement to meet a researcher. One participant opted to be interviewed with their support worker and one participant agreed for their interview to be used for analysis only (no verbatim quotation). Interviews lasted up to two hours, with one conducted over two meetings.

Participants were mostly white men living in areas of high socioeconomic deprivation. Most were serving a community sentence (e.g., unpaid work). Almost all self-identified as having current mental health or substance use needs, and the majority were accessing some support. Several were seeking help, and most had at least one unsuccessful help-seeking attempt. A substantial proportion of participants’ social networks had experienced problems with their mental health, substance use, or with the criminal justice system. Most network members were perceived to be supportive of help-seeking for mental health and substance use. See Table 1. Social network members were more equally split by gender and almost half had been known to the participant for over 10 years. They were slightly older on average and similar in ethnicity. Network members were described most frequently as friends, followed by family members and service providers. Full information of network members characteristics and the variation by gender is included in S1 Table in the S1 File.

**Table 1:** Sample characteristics.

Characteristic	N = 50
**Age (years)**	39 *Range 20–65*
**Gender**	
Male	35 (70%)
Female	15 (30%)
**Ethnicity**	
White Scottish	44 (88%)
Other	6 (12%)
**Local authority**	
LA1	23 (46%)
LA2	27 (54%)
**Living in 20% most deprived area**	37 (74%)
**Criminal justice involvement type**	
Community sentence	30 (67%)
Other	15 (33%)
**Current MH/SU condition**	45 (90%)
**Has MH/SU support**	39 (78%)
**Currently help-seeking**	29 (58%)
**Previous failed help-seeking attempt**	38 (76%)
**Average proportion of network with experience of MH problems**	56%
**Average proportion of network with experience of SU problems**	45%
**Average proportion of network with experience of criminal justice system**	36%
**Average proportion of network supportive of MH help-seeking**	80%
**Average proportion of network supportive of SU help-seeking**	84%

MH = mental health, SU = substance use.

Our framework has three categories (themes): desire to seek help, ability to seek help, and help-seeking context, each with further subthemes. ‘Desire to seek help’ is conceptualised as the yearning for something different (or not) and the motivation to achieve this (or not), whereas ‘ability to seek help’ describes the extent to which participants had the skills and resources available to them to achieve their desires. Desire and ability are further divided between individual and network influences, reflecting the integral importance of relational influences. ‘Help-seeking context’ described the wider community environment in which participants lived, including physical, social and cultural elements. See Fig 1.

### Desire to seek help

Desire to seek help was a crucial component of help-seeking, which was influenced by individual and network members’ beliefs, attitudes, and perceptions, as well as external factors.

#### Individual beliefs, attitudes, and perceptions.

Participants held varied attitudes and beliefs about help-seeking and people experiencing mental health and substance use problems (including themselves), and had differing perceptions of service providers (whether statutory, third sector, professional, or peer). Together these influenced participants’ desire to seek help.

Many participants held unfavourable views about help-seeking, feeling that help-seeking was shameful or could lead to negative outcome(s).

*I have dealt with it for almost twenty odd years by myself… I don’t like going to doctors because I think they are a waste of time…. [P12]*

Participants’ attitudes and beliefs were often rooted in childhood and past experiences or influenced by others, but could change with positive later experiences.

*A lot of folk see services as taboo… They are just [there] to do a job and you are just a number. And it’s hard that folk feel like that, because it took me even a long time to realise it that they are here to help you. [P43]*

For many participants, accumulated negative experiences with professional/statutory services deterred them from help-seeking. For others, professional services felt safest.

*You feel that you’re in good hands, certainly. They’ve seen it all before. [P45]*

Several participants were sceptical about mutual aid/peer support and were hesitant to use these. Others had validating experiences from support provided by people with shared experiences, leading them to prefer peer over professional support.

*The fact that they’ve got lived experience makes it a lot better experience for me because I can talk to them one to one. Whereas if I’m going to see a health professional. It’s just another statistic to them, you know? Have a pill, take that, you’ll feel better. [P29]*

Many participants judged themselves harshly, preventing them seeking help. This was connected closely with stigma in their wider communities, returned to in the ‘help-seeking context’ theme below.

*I’m trying but it’s just I hate this, I’ve never been able to look at myself in the mirror… I hate walking out now and I’m all over the place… My heads all fried…. I’m damaged with it all… I know it’s me. [P26]*

#### Network beliefs, attitudes, and perceptions.

The beliefs, attitudes, and perceptions of network members could shape desire for help-seeking via their impact on participants’ views, and also led participants to attempt to manage how they were perceived within their networks. Network members could undermine help-seeking desire by normalising problems, sabotaging help-seeking, being thought likely to respond negatively, and by sharing negative perspectives of help-seeking and services.

*If you think about the type of person you’re dealing with, you can figure out if it’s worth asking them for their opinion. You know, or if you’ll get shot down or if they’ll give you an ear to listen to you. [P10]*

In contrast, network members speaking openly about help-seeking could challenge negative views, introduce the idea that help-seeking was acceptable, and motivate participants to seek help themselves.

*So, I’ve seen my friends take the next step, so if you can do it, so can I. [P11]*

Network members often shared attitudes and beliefs about service accessibility, availability, and utility. Network member(s) endorsement could influence which (if any) service participants approached. A few participants described anti-engagement attitudes in their networks and had heard about network members’ negative experiences. This contributed to participants dismissing or avoiding help-seeking. For example, common phrases among people accessing substance use mutual aid programmes suggested shared attitudes and beliefs in the networks around those programmes. Many emphasised the superiority of ‘lived experience’ and dismissed professional services, where staff were perceived to have inferior ‘book learning’.

*I would listen to somebody more that’s been through it and done it and go through the other side of it … Rather than somebody that’s reading books and stuff like. [P46]*

This could prevent network members from knowing the potential benefit of certain services, or lead to avoidance out of a desire to preserve their reputation and relationships.

Because of the way participants wanted to be perceived in their network, help-seeking necessitated management of their projected identify. Several participants perceived their network to value strength and autonomy, which deterred help-seeking. When participants considered help-seeking, they had to contemplate status-loss and isolation.

*If I was sitting asking for help, I would look weak. And, at least, if I was committing crimes. That’s what I’ll do. Oh, he’s still a badass. He’s nae asking for help. He’s being forced to work with them. [P1]*

#### External events.

For some participants, their decision to seek help was triggered by external events, including experiencing a crisis (e.g., emergency admission), exhaustion, or the death of network members.

Crises in participant’s lives were often reported as moment of increased desire to change. Those most often discussed were health-related emergencies, such as being revived following an accidental overdose or being hospitalised following a suicide attempt. These experiences left participants feeling without alternative options and more open to seeking change.

*it took me until I tried to hang myself and ended up getting help again. ... I went to the doctor and said I need help [P21]*

Other participants reported a loss of desire for their lifestyle, contributed to by physical and psychological fatigue, living for a prolonged period in stressful and impoverished circumstances, or persistent victimisation. They were aware that this differed from what others in their networks had achieved, and were motivated to seek help by feeling unable to continue otherwise.

*learned to ask for help because I’m very, very tired of getting hurt all the time. [P49]*

Several participants described the impact of deaths within their social networks on their help-seeking. For some death of a loved one could encourage self-appraisal and desire to change. The death(s) of network members with similar mental health challenges or using substances could throw participants’ own vulnerability into sharp relief. Participants acknowledged the emotional impact on others when a death occurred, and did not want to cause that for others. Deaths could act as a ‘reality check’, confronting participants with the potential consequences of continuing without change, and prompting action.

*Everyone around you is dying. It’s time to do something. [P38]*

However, many participants talked about experiencing external events without it resulting in help-seeking, suggesting a change in beliefs, attitudes and perceptions was also needed, alongside the skills and resources to enact sustained change.

### Ability to seek help

Whilst participants talked of their desire to seek help (or not) this was not sufficient for them to initiate help-seeking. Help-seeking was also influenced participant’s ability to seek help, contributed to by their individual abilities and capacity, and the facilitative or inhibitive resources and norms available in their social network.

#### Individual ability and capacity.

Help-seeking was influenced by participants’ abilities and capacity for action including their ability to meet services’ demands, the extent to which they saw change as their responsibility, if they felt helpless or feared the consequences of help-seeking, and their coping styles.

Many services were thought to have strict criteria for access that the participants didn’t believe they could meet, including requirements to attend at inflexible appointment times, behave in certain ways participants, have sufficiently severe difficulties, and be substance-free to start mental health treatment. Some services delivered support in groups, which deterred many participants.

*You would go to a substance misuse with the hope of an appointment and then you would get someone telling you, oh, you can’t do this, and you can’t do that. I’m like, well, you know, you’re not the person taking the drugs. You don’t know what that’s life like. [P2]*

Some participant’s had ‘lost faith’ that they would ever be able to access services and ultimately be helped, leading them to stop trying. This lack of confidence, combined with difficulties to meet service demands for sustained commitment, eroded hope that life could be different.

*I’ve tried everything for a few times now.... it’s as if they don’t know what my illnesses are. It’s hard to even ask them for help... They don’t understand what’s going on [P26]*

Some participants feared what would happen if they didn’t seek help. Others expressed fears around help-seeking, including judgement or rejection, being denied a service, negative consequences for access to medication or access to their and children (women and men), and about what would be required of them.

*The whole stress thing, I think talking to someone face to face is a big thing... I just couldn’t go in… My whole body kind of froze, I thought, no, I’m not ready for this. [P11]*

Several participants believed that they were autonomous decision-makers with personal responsibility for change. However, taking responsibility required people to reach a point of readiness. When that point came, most participants still needed to activate the knowledge of people in their network in the course of help-seeking.

*I had to get things myself, try to get help like going to speak to a doctor and that. [P23]*

Several participants talked about how they coped with distress using drugs or alcohol rather than seeking help, often because this is what they had learned from their networks.

*I grew up in an addictive household and all… I just thought that was kind of normal to just mask your feelings and how you felt and use drugs and drink. [P46]*

Similarly, many described that were influenced in childhood to cope by concealing distress and portraying themselves as strong or independent.

*That’s more a childhood thing from when we were brought up, if you asked for anything, you got a beating. So, the best way is not to ask. It just seems that’s the way I am as an adult, I’d rather do it myself or I never get it. [P21]*

#### Network culture and resources.

People’s ability to seek help was further enabled or constrained by relational factors, including the resources available to them in their network and the collective network norms around mental health, substance use and help-seeking, which moved beyond individual network members’ beliefs, attitudes and perceptions, to describe participants’ perceptions of more generalised community norms. Culture, resources and norms were shaped by network structure.

For many participants, lack of emotional, practical, and informational resources hindered help-seeking. Several described how an absence of emotional support from caregivers as children had shaped their ability to trust that help would be forthcoming. For many, family relationships continued to be unsupportive. Experiences of service providers failing to provide a supportive context also had a long-term impact on participants’ ability to seek help from services.

*…can’t get into surgery, can’t get help. And when you do, you’re just undressed with your eyes as soon as you go in. So, it just puts you off going and asking for it, you know...[P8]*

Developing trust that a person would be supportive took time and required network members (including service providers) to demonstrate reliability. Examples included starting with practical assistance before being perceived as a safe person to seek mental health or substance use-related help from. For several participants, developing sufficient trust to seek help was hampered by experiences of network members who took resources from them to undermine help-seeking, rejection, and fear of losing the little support they had.

*I know he’s going to rub it in my face if I am not, so what do I do? Do I go home and be alone or do I stay and just, my head is fucked. [P15]*

Almost all participants perceived their network members to lack knowledge about mental illness or when substance use became problematic. Thus, they did not see difficulties arise, prompt someone to get help, know how to help, or know where to direct participants. Network members who had knowledge to share, or were willing to find it, could facilitate help-seeking when a participant could accept and act on that help.

*because I don’t know how to go about it because I will be asking them for help and they can’t help me [P13]*

Collective norms within networks shaped what was perceived to be problematic, what was an acceptable response, and what the consequences of help-seeking may be. Many participants described how mental ill-health and substance use was common in their networks leading to under-recognition of problems.

*there is a lot of people who have mental health don’t speak about it and they just get worse on drugs. [p13]*

How participants perceived their network members to respond to their own problems shaped participants’ perceptions of help-seeking.

*Dad has had a drinking problem for a long time and he just doesn’t like authority, you know what I mean? He doesn’t like the idea [help-seeking] cause he thinks you’ll be sectioned [detained under mental health legislation] or something like. [P39]*

Participants who had committed certain offences, particularly sexual offences, were acutely aware of their vulnerability to retaliation for breaching accepted network norms, even among others who had committed offences. Concealing their offending history to protect what little they had made help-seeking very challenging, especially in small communities.

*If I mentioned that I could lose the one thing that’s basically getting me help right now. [P3]*

Whilst resources and norms existed within networks, how these flowed to the individual to influence their help-seeking varied depending on the structure of their network. Several participants described how their network size and connections could impact help-seeking by constraining or enabling access to resources and the way norms were embedded. Small, closed networks were described as presenting risks in relation to access to diverse thought, opinion, and resources, or because information and negative appraisals could spread easily.

*We’re such a small town, if there is any groups on the go, people feel they can’t go to them because people know their families. [P7]*

A few described the benefits of a diverse network for exposure to people taking steps to address mental health and substance use challenges. However, for many, large networks resulted in more people disrupting their attempts to change.

*you just kind of can’t get away from the social, every time you try and do something good basically they will get you on it and you will be wrecked and miss what you are going to do. [P13]*

Most participants had restructured their networks, often deliberately removing people to facilitate change. Some participants restructured networks to remove those who encouraged help-seeking, but most described changes to remove those perceived as negative influences.

*I just started cutting people off. I think people were used to a certain version of me, and once I was working on that version of me trying to change, people just didn’t appreciate the self-development… I started realising that they weren’t friends. [P10]*

Regardless of individual capacity and network norms and resources, participants acknowledged that there were realities outside this that had a further impact on help-seeking.

### Help-seeking context

All participants were cognisant of the context in which they were seeking help. This included the realities of services in their areas; factors specific to their locality; the local culture (beyond their immediate network) around help-seeking; and network and societal stigma towards mental health, substance use, and help-seeking.

Several participants perceived service providers, particularly statutory health and social care services, as working with caseload demands beyond their capacity, meaning help-seeking seemed futile. Despite many participants describing statutory services as well-intentioned, several disclosed poor experiences that deterred help-seeking.

*I was crying on the phone and then they said there was nothing they could do… So I got turned away and my faith was just lost in them. [P29]*

The reality of availability and accessibility of services, particularly in LA1 where services were dispersed over a large geography, required substantial commitment to make contact. Further, participants were conscious that anonymity was unlikely and confidentiality uncertain in small communities, and were hesitant to help-seek in these contexts.

*everybody knows you and that’s maybe why I go outwith the town. [P16]*

Most LA2 participants held positive views of the third sector and peer support services. However, several had poor experiences with staff or processes that deterred help-seeking. This was attributed to over-confidence that people with lived experience could understand all experiences, being exposed to unhelpful others, and funding models that led to prioritisation of meaningless indicators.

*I don’t think it’s a good idea because you can, like when you’re with certain groups and all, you get led astray. … right after the group thing they go “You want to go for a rock? [P34]*

Though such services were scarce in LA1, some participants had similar varied perceptions about what these *would* be like.

Several participants described how the prevalence and visibility of problems, particularly of drug use, made help-seeking harder. Temptation and people who condoned and enabled guilt-free drug use were everywhere. Some participants believed recent public consciousness about the harms of drugs had driven political and local efforts to improve substance use services, though this was not seen for mental health.

*It is different here [large town]. But because of the deaths and the struggles here. People have decided to get their finger out about it. [P36]*

Others described how the local culture (particularly in LA1) did not support help-seeking and strongly stigmatised being unable to cope independently. The associated shame and fear of exposure in the community as ‘weak’ deterred them from help-seeking.

*But for me to ask for someone’s help it’s a sign of weakness and it makes me feel like I am weak, I feel numb. And I just want to put on a brave face and go out with a brave face on and say like everything is okay. [P12]*

Further, most participants perceived and experienced societal- and network-level stigma towards people with mental health and substance use problems. Several participants were stigmatising towards themselves and others. For a few, not wanting to “*end up like that” [P4]* (long-standing substance use or mental health problems/a life of repeated imprisonment) motivated help-seeking. But for most, being identified within a stigmatised group deterred help-seeking, that was heightened in cultures where help-seeking was viewed as a sign of inadequacy.

## Discussion

We present findings from the first in-depth qualitative study into the influences on help-seeking for mental health and substance use among people in contact with the criminal justice system in the community. Rigorous qualitative data analysis of 50 interviews undertaken within a mixed-methods social network analysis study, confirmed that desire to seek help, ability to seek help and help-seeking context are important influences on help-seeking in this population, consistent with theories of human behaviour [14] and early healthcare access models [10]. Our attention to social networks, in addition to individual and contextual factors, revealed the importance of relational influences operating across participants’ social networks and communities. This is a previously under-explored topic and thus, our findings make threesignificant contributions. First, we have advanced understandings of the factors influencing help-seeking for mental health and substance use among people in contact with the criminal justice system, particularly highlighting the need for nuanced appreciation of relational and contextual influences beyond the individual. Second, we make a methodological contribution to research examining behaviour and behaviour change with under-served/marginalised populations by highlighting the enhanced understanding attained by explicitly asking about peoples’ social networks in their distinct communities. Third, the framework provides a resource to inform the design and delivery of individual, social network, community-level, and public health interventions to optimise mental health and substance use-related help-seeking among people in contact with the criminal justice system living in the community. This could ultimately lead to more people securing timely access to appropriate services that reduce the risk of health crises or avoidable deaths.

The importance of individual knowledge, attitudes, beliefs, and perception of stigma are well-documented as influencing help-seeking for mental health and substance use [18–22], including among people in prison [45]. Demonstrating the relevance of these factors in a population involved with the criminal justice system in the community supports applying established cognitive-behavioural models to explain mental health and substance use-related help-seeking within this population [14,46]. However, our findings highlight the importance of areas that are only beginning to be considered in healthcare access models [10], and the potential limitations of considering help-seeking as a rational, individually-driven choice [43,47]. If we are to fully appreciate the factors influencing mental health and substance use-related help-seeking among people in contact with the criminal justice system, focused attention on relational influences operating across social networks and communities is required.

Social networks shape behaviour by providing (or withholding) resources, information and emotional support, and by subtly (or not) enforcing attitudinal and behavioural norms [48–51]. Perry and Pescosolido [43] demonstrated that average network-level trust in service providers was associated with mental health help-seeking. Our findings support this by qualitatively illustrating how the inverse, network *mistrust* of service providers can deter help-seeking. Epistemic mistrust is rooted in negative experiences in early relationships and is characterised as a self-protective position of dismissing others as unlikely to be safe information recipients/holders, to really care, or to be able to offer anything useful [52]. Adverse and abusive childhood experiences are associated with mistrust of others and thus avoidance of help-seeking among people sentenced to imprisonment [53]. We corroborate this in a large community sample and highlight how, not only services, but the knowledge they hold and their potential to be useful was mistrusted across peoples’ networks, further entrenching norms around rejecting help-seeking. Our findings indicate that epistemic mistrust, whilst often understood individually, can be shared across social networks. Thus, developing the trust needed to facilitate help-seeking for stigmatised conditions in under-served communities will likely require community-level cultural change.

A culture that characterises help-seeking as an indicator of weakness, beyond the stigma towards experiencing problems in the first place, has been shown to influence mental health help-seeking in other populations [54], but there are particular considerations when people are also involved in the criminal justice system. The need to project strength and autonomy to protect oneself from potential adversaries is articulated by men in prison [55]. In our community setting there was less emphasis on physical survival, and greater concern with community rejection, but both populations seek to preserve their ‘strong’ identities. Ramesh et al. [55] described a need to project strength as associated with the prison environment’s demands to perform masculinity. However, the presence of this in our community sample, and amongst women, suggests this may reflect common intergenerational norms in the networks of people in contact with the justice system. That is, norms that mental illness, substance use problems, and help-seeking are viewed as weakness.

Increasing public understanding of mental health and substance use problems and destigmatising help-seeking is an ongoing aim of public health campaigns in Scotland and elsewhere [56,57]. Despite participants’ networks being characterised by high levels of mental health and substance use problems, knowledge about mental health and substance use problems and how to address these was limited. This suggests that traditional strategies of information dissemination and anti-stigma messaging may fail to reach those in greatest need, potentially linked to the shared epistemic mistrust of information providers. Co-produced communication campaigns that capitalise on local culture and use trusted credible messengers and common communication formats, may be more likely to have success in increasing knowledge, problem recognition, and awareness of available support [58]. However, additional investigation of the mechanisms of influence and how best to increase knowledge are first needed.

Health care is free at the point-of-use and funded through general taxation in Scotland, so despite availability challenges, cost was not identified as a contextual influence as it may be in other countries. Nonetheless, access barriers remain. Scotland has seen substantial investment in support and treatment for illicit drug use in recent years [59]. However, mental health services remain oversubscribed, inadequately resourced, difficult to navigate, and often exclude people with substance use problems [60,61]. This remains problematic for people in contact with the criminal justice system where experiencing both mental health and substance use problems is common [62]. Given the individual and public health implications of unmet mental health and substance use needs among people in contact with the criminal justice system, it is vital that if people are encouraged to seek help, their contemporary experience is positive. Mental health and substance use services need to be adequately resourced to meet the level of need, and skilled to overcome the collectively remembered negative experiences in this population. Trauma-informed and relational practice approaches are likely to be an important component of more positive experiences [63,64]. Further, services need to be able to accommodate people who cannot meet inflexible demands and strict expectations. Services that take an inclusion health approach [65] may be beneficial, with consideration for remote, rural, and urban contexts, though evidence for the impact of inclusion health approaches is still emerging.

Intervention could be shaped by the framework we have developed. Service providers could utilise the framework to identify which influences may be evident in client’s life, and tailor intervention accordingly. However, the framework should be applied critically, and not be considered definitive at this stage. Future research could further refine and confirm the frameworks utility; identify which factors, in which combinations, are most modifiable and have the greatest impact for different groups in different contexts; and examine the mechanisms by which different factors have their effects on help-seeking. There is potential to use the framework to design and evaluate multi-level complex interventions to increase mental health and substance use-related help-seeking among people in contact with the criminal justice system, and to understand how interventions may need to vary across individuals, networks, and contexts. The framework can also be tested for utility in understanding the needs of individuals experiencing other stigmatised issues or from other marginalised groups, and designing intervention to encourage help-seeking.

## Strengths and limitations

Our study has several strengths including a large sample size, recruitment from two distinct geographical areas that highlighted contextual influences, rigorous team analysis, and involvement of people with lived experience in the research team and LEAP. We published protocols a-priori [39]. Our framework was developed with a focus on mental health and substance use among people in contact with the criminal justice system. Given the common intersections between criminal justice system contact and other stigmatised experiences (e.g., homelessness) [66], our framework has potential to explain help-seeking for other stigmatised issues (e.g., HIV/AIDs, victimisation/abuse) in other marginalised populations. Further research would be beneficial to confirm this.

There are four limitations. Firstly, recruiting people who were not already receiving or seeking mental health or substance use support was challenging, due to our primary recruitment strategy being via service providers. However, many participants reflected on past experiences, providing insights into their experience when not seeking or accessing help. This was successful in counterbalancing any potential ‘pro’ help-seeking bias, and rich accounts are evident in the data. Secondly, we did not recruit in a city. Whilst the influences on help-seeking in cities may differ, our between-context comparison allowed us to identify context-specific factors that can be considered in any setting. Thirdly, distinguishing individual, social, and contextual influences on a complex human behaviour was challenging. Readers and those seeking to apply the framework in research and practice should be mindful of its preliminary nature and potential for overlap in the realities of peoples’ lives. Finally, caution will be needed in considering transferability of our findings to countries with different demographic characteristics, and legal, criminal justice, health, and social services. For example, in more ethnically diverse communities, intersecting stigma may have an additionally deterrent effect, or in countries where health insurance is required, cost/registration challenges would also need consideration.

## Conclusion

Facilitating early mental health and substance use help-seeking among people in contact with the criminal justice system is an important public health concern, with potential to reduce later needs for emergency intervention and avoidable mortality. Our research demonstrates that the desire and ability to seek help among people in contact with the criminal justice systems are considerably influenced by relational factors, operating across social networks embedded in distinct local and national contexts. This highlights the advantages of taking a deliberately social and relational perspective in understanding help-seeking, particularly in relation to stigmatised conditions among underserved communities.

Whether working at individual, social network, community or a wider societal level, the framework produced can aid in identifying intervention targets for increasing help-seeking among people in contact with the criminal justice system. The complex relationship between individual, relational, and contextual influences indicates that multi-level interventions may be needed and that these should be further tailored to specific individuals, social networks, and localities.

## Supporting information

S1 File Supplementary file. S1 Table. Characteristics of network members. S2 Table. Definitions of themes and codes.
S2 Table. Definitions of themes and codes.
(DOCX)
