# Supplementary material for: You “can’t get away from the social”: Individual, relational, and contextual influences on mental health and substance use-related help-seeking among people in contact with the criminal justice system
Source: PLoS One. 2026 Jul 30;21(7):e0354996. doi: 10.1371/journal.pone.0354996 (PMC13422872; doi:10.1371/journal.pone.0354996)
Supplement: S1 File — S1 Table. Characteristics of network members. S2 Table. Definitions of themes and codes. S2 Table. Definitions of themes and codes. (DOCX) [file pone.0354996.s001.docx]

# Supplementary file

S1 Table: Characteristics of network members.

|  | **Female (N=211)** | **Male (N=238)** | **Total (N=449)** | **p value** |
| --- | --- | --- | --- | --- |
| **Age in years** |  |  |  | 0.9 |
| Mean (SD) | 42.9 (16.5) | 43.1 (14.1) | 43.0 (15.3) |  |
| Range | 1 - 84 | 2 - 77 | 1 - 84 |  |
| **Ethnicity** |  |  |  | 0.4 |
| White Scottish | 181 (85.8%) | 212 (89.1%) | 393 (87.5%) |  |
| White other | 24 (11.4%) | 20 (8.4%) | 44 (9.8%) |  |
| Other | 6 (2.8%) | 6 (2.6%) | 12 (2.6%) |  |
| **Relationship to participant** |  |  |  | < 0.001 |
| Family | 80 (37.9%) | 62 (26.1%) | 142 (31.6%) |  |
| Friend | 50 (23.7%) | 108 (45.4%) | 158 (35.2%) |  |
| Worker | 60 (28.4%) | 47 (19.7%) | 107 (23.8%) |  |
| Partner | 8 (3.8%) | 7 (2.9%) | 15 (3.3%) |  |
| Acquaintance | 8 (3.8%) | 8 (3.4%) | 16 (3.6%) |  |
| Colleague | 2 (0.9%) | 5 (2.1%) | 7 (1.6%) |  |
| Other | 3 (1.4%) | 1 (0.4%) | 4 (0.9%) |  |
| **Duration of relationship*** |  |  |  | 0.0 |
| 0 - 6 months | 20 (9.5%) | 20 (8.4%) | 40 (8.9%) |  |
| 6 months - 1 yr | 21 (10.0%) | 38 (16.0%) | 59 (13.2%) |  |
| 1 - 5 years | 58 (27.6%) | 37 (15.5%) | 95 (21.2%) |  |
| 5 - 10 years | 16 (7.6%) | 20 (8.4%) | 36 (8.0%) |  |
| 10 years | 95 (45.2%) | 123 (51.7%) | 218 (48.7%) |  |
| **Frequency of contact*** |  |  |  | 0.3 |
| Daily | 69 (32.9%) | 86 (36.3%) | 155 (34.7%) |  |
| Weekly | 101 (48.1%) | 94 (39.7%) | 195 (43.6%) |  |
| Monthly | 23 (11.0%) | 35 (14.8%) | 58 (13.0%) |  |
| Less often | 17 (8.1%) | 22 (9.3%) | 39 (8.7%) |  |

**One case missing data excluded, **two cases missing data excluded*

S2 Table: Definitions of themes and codes.

| **Desire to seek help – motivation to make change and seek help to make it happen** |  |
| --- | --- |
| **Individual beliefs attitudes and perceptions** | How individuals’ beliefs and attitudes towards, and perceptions of mental health, substance use, help-seeking and services contribute to motivation to make change and seek help.  Strongly influenced by networks and contexts |
| **Beliefs and attitudes** | Individuals’ beliefs and attitudes towards mental health, substance use, and help-seeking. |
| **Views of service providers** | Individuals’ perceptions of services providers and their responses to help-seeking. |
| **Internalised stigma** | Individuals’ negative self-appraisal for having mental health or substance use problems and being unable to resolve these independently. |
| **Network beliefs, attitudes and perceptions** | How social network members’ beliefs and attitudes towards, and perceptions of mental health, substance use, help-seeking and services influenced participant motivation to make change and seek help.  Directly influenced help-seeking desire, but also influenced participants’ ‘individual’ beliefs attitudes and perceptions which then influenced desire |
| **Beliefs and attitudes** | Social network members’ beliefs and attitudes towards, and perceptions of mental health, substance use, help-seeking, services and the participant. |
| **Views of service providers** | Social network members’ perceptions of services and their responses to help-seeking. |
| **Social identity** | Social network perceived the participant with a certain identity in relationships/the network. Wishing to sustain/change this perception and social identity. |
| **External events** | Events within participants’ lives that had an impact on their perception of their position and potential/need to seek help |
| **Crisis** | Crisis motivated help-seeking to facilitate survival. |
| **Maturing / burning out** | Exhaustion/feeling constrained by with living with mental health and substance use. |
| **Network deaths** | Confrontation with mortality of self and others and regrets where relationship quality deteriorated. |
| **Ability to seek help** | **- the skills and resources available and accessible that make help-seeking possible** |
| **Individual ability and capacity** | How individuals’ skills and the capacity to use them influence help-seeking |
|  |  |
| **Meeting (service) demands** | Knowing about, being able to meet, and believing they had the ability to meet service expectations for help-seeking (e.g. online forms), and engagement influenced help-seeking. |
| **Helplessness** | Failed attempts at help-seeking and change leading to a position of apathy. |
| **Fear** | Fear of the response (from social network and services) to help-seeking attempts and of the changes that may be requested on engagement. |
| **Responsibility** | Responsibility to seek/offer help where people do/do not have the ability. |
| **Coping style** | Individual ability to cope with challenges, including to identity, when help-seeking. |
| **Network barriers and facilitators** | How the structure, culture and resources held within a network that influence help-seeking.  Influenced help-seeking ability, but also influenced participants’ ‘individual’ abilities/capacities which then influenced |
| **Resources** | How networks provide support and knowledge that enable or constrain help-seeking |
| **Support** | Emotional support and tangible assistance (or lack thereof) available via the network.  Individuals’ trust that network members would be able to provide support if asked, without harm to either party. |
| **Knowledge** | Accurate and trustworthy information and advice (or lack thereof) available through the network.  Underpinned by (lack of) of mental health and substance use awareness/knowledge across the network. |
| **Network norms** | How network norms and cultures (distinct from wider communities) influence participants ability to seek help |
| **What is considered a mental health or substance use problem** | The extent to which living with mental ill-health and harmful substance use is normalised in network. Linked to knowledge of what is help is available for. |
| **View of (engaging with) authority** | Perceptions of the motivations of state provided services, and the extent to which engaging with structures and services are approved of. |
| **Vulnerability from offence types** | Network views of offences and calculating the risk of harm to self and others through exposure when help-seeking. |
| **Structure** | How the size, inter-connectedness, composition, and durability of a network can influence help-seeking |
| **Help-seeking context** | Influences on help-seeking from beyond the immediate network |
| **Stigma** | Perceived stigma towards mental health and substance use beyond the immediate network (local community, societally) influenced willingness to seek help. This acted on help-seeking desire and ability, though immediate networks were more influential. |
| **Realties of service availability** | The extent to which there are services available to seek help from influenced help-seeking. |
| **Geographical factors** | The extent to which services were accessible in the context of the persons locality to seek help from. |
| **Local culture** | The extent to which help-seeking was seen as indicative of strength/weakness or socially desirable, beyond the immediate network. |
