# Supplementary material for: You “can’t get away from the social”: Individual, relational, and contextual influences on mental health and substance use-related help-seeking among people in contact with the criminal justice system
Source: PLoS One. 2026 Jul 30;21(7):e0354996. doi: 10.1371/journal.pone.0354996 (PMC13422872; doi:10.1371/journal.pone.0354996)
Supplement: Attachment — Submitted filename: Reviewer comments.docx [file pone.0354996.s002.docx]

**You “can’t get away 1 from the social”: Individual, relational, and contextual influences on mental health and substance use-related help-seeking among people in contact with the criminal justice system**

**ABSTRACT**

The abstract reads descriptive, not conceptually innovative. Explicit theoretical contribution and why findings change existing knowledge were missed. Add: Conceptual gap, theoretical advancement, mechanism explanation and Broader implications.

**Overall Evaluation**

This manuscript presents a qualitative investigation of help-seeking behaviour among individuals in contact with the criminal justice system (CJS) living in community settings.

However, several conceptual, methodological, and presentation issues limit the manuscript’s current contribution. Most importantly:

- The theoretical positioning requires stronger integration and originality clarification.
- The analytical framework risks appearing descriptive rather than explanatory.
- The link between social network analysis and qualitative findings needs clearer articulation.
- Some methodological decisions require deeper justification.

**Theoretical Contribution Needs Clearer Positioning**

The manuscript draws from:

- help-seeking models,
- healthcare access frameworks,
- social network theory,
- stigma literature.

However, the novel theoretical contribution remains insufficiently articulated.

Currently, the “desire–ability–context” framework resembles existing access models (e.g., Andersen’s Behavioural Model, Levesque et al.’s healthcare access framework). The question is**:** What is fundamentally new here?

The authors should explicitly clarify:

- Whether this framework is: an adaptation, extension, synthesis, or critique of existing models.
- How relational/network mechanisms advance theory, not only empirical understanding.

**Underdeveloped Integration of Social Network Analysis (SNA)**

The study is described as a mixed-methods social network analysis study, authors should:

- Clarify how SNA informed analysis (not only data collection)
- Provide examples linking network properties to themes
- Consider integrating visual/network findings into Results

Otherwise, the SNA framing risks appearing overstated.

**Analytical Depth: From Description to Mechanism**

The findings richly describe influences but less clearly explain mechanisms. Consider framing mechanisms such as: identity preservation, social surveillance, normative reinforcement, emotional resource exchange, reputational risk.

This would significantly increase theoretical sophistication.

**Conceptual Overlap Between Themes**

The categories desire, ability, and context occasionally overlap conceptually. Author should provide: clearer operational definitions, a conceptual diagram, explanation of boundaries between themes.

Readers should understand why a finding belongs to one category rather than another.

**Sampling and Transferability**

Expand discussion on: structural welfare context, justice system differences internationally, implications for diverse populations.

**Reflexivity Section is Strong but could be Analytically Used**

The reflexivity statement is commendably transparent but remains procedural.

The authors should discuss: how researcher positionality shaped interpretation, interactions with marginalized participants, power dynamics during interviews.

Reflexivity should inform analysis, not only reporting compliance.
