# Supplementary material for: You “can’t get away from the social”: Individual, relational, and contextual influences on mental health and substance use-related help-seeking among people in contact with the criminal justice system
Source: PLoS One. 2026 Jul 30;21(7):e0354996. doi: 10.1371/journal.pone.0354996 (PMC13422872; doi:10.1371/journal.pone.0354996)
Supplement: Attachment — Submitted filename: Comments.docx [file pone.0354996.s003.docx]

Overall, this is an interesting, relevant, and clearly structured manuscript addressing an important topic. Its main strength lies in showing that help-seeking among people in contact with the criminal justice system cannot be understood as an individual decision alone, but is shaped by relational, network-based, and contextual influences. The qualitative material is rich and the focus on social networks has clear potential. However, the manuscript would benefit from a somewhat stronger theoretical positioning in relation to existing help-seeking and access models, a more integrated analytical account of how individual, relational, and contextual factors interact, and a more explicit reflection on the implications of recruiting mainly through services for the interpretation of the findings.

**Additional comments**

- **Introduction:** Some additional information on the existing state of research would be helpful, especially to clarify more precisely what is already known and where the present study adds new knowledge.
- **p. 3, lines 3–4:** More specific information on the types of mental health problems common in this population would be useful.
- **p. 3, lines 17–20:** Please specify what kinds of insights general population surveys provide. The current wording remains rather broad.
- **p. 3, line 24:** Typo: “In health researche.e.g.” should be corrected.
- **p. 5, lines 15–16:** Please clarify what is meant by excluding participants if participation “may negatively impact their health, sentence completion, or researcher safety.” Who assessed this, and according to which criteria?
- **Methods:** Please clarify how it was determined whether participants had mental health or substance use problems. Was this self-reported, assessed through service records, screening instruments, or interview data?
- **p. 6, line 18:** A word seems to be missing.
- **p. 7, Research team and reflexivity:** The qualifications of the research team are described later, but the allocation of specific tasks and expertise to individual team members could be made clearer.
- **p. 7, line 26:** Please clarify what is meant by “personal and professional experience of the criminal justice system, mental ill-health, and substance use problems.” This sentence is potentially ambiguous and may require more careful phrasing.
- **p. 8, line 1:** There appears to be one superfluous word: “on.”
- **p. 8/Table 1:** In order to understand the table, more information on the social network analysis would be helpful. What types of networks were captured, how extensive were they, and how were they elicited? The manuscript refers to the interview protocol, but this was not included in the review materials.
- **p. 10, line 20:** Repetition of the word “seeking.”
- **p. 19, line 24:** Repetition of the word “first.”
- More information on participants’ networks would strengthen the paper overall. Were these networks predominantly characterised by similarly disadvantaged socio-economic and cultural backgrounds? Were some networks more supportive or more destructive than others? Which participants reported more positive experiences with psychosocial help, and did this relate to network composition? Were there any gender differences in the accounts?
- **Figure 1, p. 37:** The image quality is poor, and the category system is not fully self-explanatory. Clearer category labels would be useful, for example “Desire to seek help” rather than simply “Desire.”
