# Supplementary material for: You “can’t get away from the social”: Individual, relational, and contextual influences on mental health and substance use-related help-seeking among people in contact with the criminal justice system
Source: PLoS One. 2026 Jul 30;21(7):e0354996. doi: 10.1371/journal.pone.0354996 (PMC13422872; doi:10.1371/journal.pone.0354996)
Supplement: Attachment — Submitted filename: Response to reviewers.docx [file pone.0354996.s005.docx]

**Dr Catriona Connell** PhD. MSc. BSc(Hons). FHEA

Senior Research Fellow

**Faculty of Social Sciences**

University of Stirling, Stirling FK9 4LA

catriona.connell@stir.ac.uk[catriona.connell@stir.ac.uk](mailto:catriona.connell@stir.ac.uk)

12^th^ June 2026

Dear Editors,

**You “can’t get away from the social”: Individual, relational, and contextual influences of mental health and substance use-related help-seeking among people in contact with the criminal justice system**

Thank you for the opportunity to revise our manuscript based on the helpful reviewers’ comments. We are confident that this has further strengthened our paper. We have appended the point-by-point response

Yours sincerely,

**Dr Catriona Connell**

Senior Research Fellow | Salvation Army Centre for Addiction Services and Research | University of Stirling

Email:    catriona.connell@stir.ac.uk

Orcid:    [0000-0002-4016-5120](https://eur03.safelinks.protection.outlook.com/?url=https%3A%2F%2Forcid.org%2F0000-0002-4016-5120&data=05%7C02%7Ccatriona.connell%40stir.ac.uk%7C0fb9c93cdcef4e4c669408dc63835ac1%7C4e8d09f7cc794ccb9149a4238dd17422%7C0%7C0%7C638494663017462996%7CUnknown%7CTWFpbGZsb3d8eyJWIjoiMC4wLjAwMDAiLCJQIjoiV2luMzIiLCJBTiI6Ik1haWwiLCJXVCI6Mn0%3D%7C0%7C%7C%7C&sdata=DmKc8ONvGPZfz2w4FGVmdgHKwEg5Nozgdu53EC0oP8Y%3D&reserved=0)

**Point- by point response**

|  | **Section** | **Comment** | **Response** | **Page** |
| --- | --- | --- | --- | --- |
| Editor | Technical | Please ensure that your manuscript meets PLOS ONE's style requirements, including those for file naming. The PLOS ONE style templates can be found at  https://journals.plos.org/plosone/s/file?id=wjVg/PLOSOne_formatting_sample_main_body.pdf and  https://journals.plos.org/plosone/s/file?id=ba62/PLOSOne_formatting_sample_title_authors_affiliations.pdf | Manuscript modified to reflect style requirements | N/a |
| Editor | Technical | Please provide additional details regarding participant consent. In the ethics statement in the Methods and online submission information, please ensure that you have specified (1) whether consent was informed and (2) what type you obtained (for instance, written or verbal, and if verbal, how it was documented and witnessed). If your study included minors, state whether you obtained consent from parents or guardians. If the need for consent was waived by the ethics committee, please include this information. | Ethics section in manuscript and online system updated | 8 |
| Editor | Technical | “There was no additional external funding received for this study.” in your updated Funding Statement.  Please include your amended Funding Statement within your cover letter. We will change the online submission form on your behalf. | Updated in letter and in online system | N/a |
| Editor | Technical | Thank you for stating [... ] in the Competing Interests section: Please confirm that this does not alter your adherence to all PLOS ONE policies on sharing data and materials, by including the following statement: "This does not alter our adherence to PLOS ONE policies on sharing data and materials.” (as detailed online in our guide for authors http://journals.plos.org/plosone/s/competing-interests). If there are restrictions on sharing of data and/or materials, please state these.   Please include your updated Competing Interests statement in your cover letter; we will change the online submission form on your behalf. | See cover letter. | N/a |
| Editor | Technical | We note that this data set consists of interview transcripts. Can you please confirm that all participants gave consent for interview transcript to be published?  If they DID provide consent for these transcripts to be published, please also confirm that the transcripts do not contain any potentially identifying information (or let us know if the participants consented to having their personal details published and made publicly available).  Or, if the participants DID NOT provide consent for these transcripts to be published ...   If there are ethical, legal, or third-party restrictions upon your dataset, you must provide all of the following details (https://journals.plos.org/plosone/s/data-availability#loc-acceptable-data-access-restrictions):  1. A complete description of the dataset 2. The nature of the restrictions upon the data (ethical, legal, or owned by a third party) and the reasoning behind them 3. The full name of the body imposing the restrictions upon your dataset (ethics committee, institution, data access committee, etc) 4. If the data are owned by a third party, confirmation of whether the authors received any special privileges in accessing the data that other researchers would not have 5. Direct, non-author contact information (preferably email) for the body imposing the restrictions upon the data, to which data access requests can be sent | We have updated the manuscript ethics section and data access section on the online submission system as follows:   Participants consented to the use of their pseudonymised data for verbatim quotation (49/50) and for use in future research within the UK and European Union. The data set [and accompanying code] is available at the UK Data Service via 10.5255/UKDA-SN-858261 to support the latter.   The dataset contains pseudonymised transcripts and quantitative data collected in the wider study. These data are safeguarded owing to their sensitive nature. Approval is needed from a data access committee to access these. Ethical approval (General University Ethics Panel, University of Stirling [13947]) has been secured for deposit in the UK Data Service in line with funder requirements, and these access arrangements. TO access this dataset, please email catriona.connell@stir.ac.uk to request permission to access the data, explaining your reason for wanting access to the data, then contact the UK Data Service Access Helpdesk. | N/a |
| Editor | Technical | Please include captions for your Supporting Information files at the end of your manuscript, and update any in-text citations to match accordingly. Please see our Supporting Information guidelines for more information: http://journals.plos.org/plosone/s/supporting-information. | We have amended the manuscript and supporting information files in line with these guidelines | N/a |
| Editor | Technical | Please review your reference list to ensure that it is complete and correct. If you have cited papers that have been retracted, please include the rationale for doing so in the manuscript text, or remove these references and replace them with relevant current references. Any changes to the reference list should be mentioned in the rebuttal letter that accompanies your revised manuscript. If you need to cite a retracted article, indicate the article’s retracted status in the References list and also include a citation and full reference for the retraction notice. | The reference is complete. There are no retracted papers in the reference list. The references have been updated to reflect additions and manuscripts that have moved to 'in press'. | 25-29 |
| Editor | Technical | To ensure your figures meet our technical requirements, please review our figure guidelines: https://journals.plos.org/plosone/s/figures   You may also use PLOS’s free figure tool, NAAS, to help you prepare publication quality figures: https://journals.plos.org/plosone/s/figures#loc-tools-for-figure-preparation.   NAAS will assess whether your figures meet our technical requirements by comparing each figure against our figure specifications. | We have amended the figures to comply with PLOS One guidance | Fig 1. |
| 5 | Abstract | The abstract reads descriptive, not conceptually innovative. Explicit theoretical contribution and why findings change existing knowledge were missed. Add: Conceptual gap, theoretical advancement, mechanism explanation and Broader implications. | Revisions made to the abstract to highlight the conceptual gap, theoretical advancement (applicability of existing theory, and emphasis on relational factors), mechanisms (relationships) and implications added. We are clear that this is primarily and empirical study. | 2 |
| 4 | Intro | The manuscript would benefit from a somewhat stronger theoretical positioning in relation to existing help-seeking and access models | We have made amendments throughout to clarify our position in relation to existing models, and acknowledge/articulate the theoretical contributions | throughout |
| 4 | Intro | Some additional information on the existing state of research would be helpful, especially to clarify more precisely what is already known and where the present study adds new knowledge. | following our review of help-seekign in the prison context, we highlight that there is little known about help-seeking for people in contact with the justice system in the community, thus situating the rationale for our study. | 4 |
| 4 | Intro | p. 3, lines 3–4: More specific information on the types of mental health problems common in this population would be useful. | All types of mental health and substance use problems are common in this population. We have included some indicative figures. | 3 |
| 4 | Introduction | p. 3, lines 17–20: Please specify what kinds of insights general population surveys provide. The current wording remains rather broad. | Clarification added that this refers to associations between insights into individual factors and help-seeking | 3 |
| 4 | Intro | p. 3, line 24: Typo: “In health research e.g.” should be corrected. | Thank you for this detailed feedback. This is corrected | 3 |
| 5 | Intro | The novel theoretical contribution remains insufficiently articulated. Currently, the “desire–ability–context” framework resembles existing access models (e.g., Andersen’s Behavioural Model, Levesque et al.’s healthcare access framework). The question is: What is fundamentally new here? The authors should explicitly clarify: • Whether this framework is: an adaptation, extension, synthesis, or critique of existing models.  • How relational/network mechanisms advance theory, not only empirical understanding. | We have made amendments throughout to 1) clarify our use of published frameworks to inform desire ability and context categories, and to 2) indicate the contribution we make by more explicitly articulating relational influences. We emphasise that what is novel, is applying these frameworks with our population (who face distinct challenges) and make the links between both these empirical findings and how help-seeking theory could be discussed with greater nuance. We emphasise that this is an empirical study but highlight how relational and contextual factors may be under-articulated in existing (behaviour change/psychology) models/frameworks. | Through-out |
| 1 | Methods | I believe that within the framework analysis, the model of help-seeking behaviour and access to health services should be explained more clearly and in greater detail. | More information has been added in the data analysis section to make clear how we developed the initial framework and were sensitised to relational processes and social context. | 7 |
| 1 | Methods | I consider that the involvement of people with lived experience in the research team does not necessarily represent a strong point, as it could introduce a degree of subjectivity due to their inclusion in the research team. | Thank you for raising this point. Members of any research team bring life experiences, and potential subjectivity, irrespective of the topic. Being a researcher, especially in qualitative work, involves recognising the different perspectives and insights brought by the team appropriately. We provide detail in our reflexivity section and transparently reporting throughout using existing standards to assure readers that this is rigorous empirical work. | 8 |
| 3 | Methods | First, the methodological section would benefit from a more explicit justification of key analytical decisions. In particular, the criteria used to determine data saturation are not fully articulated, and the rationale for the final sample size could be clarified. Additionally, while the manuscript reports the use of independent coding by multiple researchers, the process for resolving discrepancies and reaching analytic consensus is not sufficiently detailed. Providing this information would strengthen the credibility and dependability of the analysis. | We have clarified how the decision to cease sampling was reached, and how sampling was informed by project resource. We reflect on this in the limitations.   We have added greater detail on the analytical process. | 5 |
| 3 | Methods | Second, the manuscript refers to a broader mixed-methods social network analysis study, yet only qualitative findings are presented. While this does not detract from the value of the study, the role of the social network component within the present analysis could be more clearly articulated to avoid potential ambiguity. At times, the framing may suggest a shift toward more quantitative or structural interpretations; however, the procedures for data collection and analysis remain clearly grounded in qualitative paradigms. Clarifying this positioning would enhance conceptual clarity and coherence. | Clarification added that this is a component of the social network study, and not a social network study itself. | 4 |
| 4 | Methods | The manuscript would benefit from a more integrated analytical account of how individual, relational, and contextual factors interact | Clarification added that these are highly inter-related and how we came to the decisions we did. | 7 & 10 |
| 4 | Methods | p. 5, lines 15–16: Please clarify what is meant by excluding participants if participation “may negatively impact their health, sentence completion, or researcher safety.” Who assessed this, and according to which criteria? | Information added to clarify how this was done. | 5 |
| 4 | Methods | Please clarify how it was determined whether participants had mental health or substance use problems. Was this self-reported, assessed through service records, screening instruments, or interview data? | In data collection we have clarified all data are self-report. | 6 |
| 4 | Methods | p. 6, line 18: A word seems to be missing. | Language clarification. | 6 |
| 4 | Methods | p. 7, Research team and reflexivity: The qualifications of the research team are described later, but the allocation of specific tasks and expertise to individual team members could be made clearer. | Added initials in more of the methods section | 6-8 |
| 4 | Methods | p. 7, line 26: Please clarify what is meant by “personal and professional experience of the criminal justice system, mental ill-health, and substance use problems.” This sentence is potentially ambiguous and may require more careful phrasing | Some additional phrasing to make this clearer. We deliberately do not allocate experiences to colleagues to protect their privacy. | 8 |
| 4 | Methods | p. 8, line 1: There appears to be one superfluous word: “on.” | Thank you for spotting this, deleted | 8 |
| 5 | Methods | Authors should: • Clarify how SNA informed analysis (not only data collection) • Provide examples linking network properties to themes  • Consider integrating visual/network findings into Results Otherwise, the SNA framing risks appearing overstated. | We have clarified that this is a qualitative study within a wider social network study, and thus, whilst sensitised to the importance of social networks and relational influences, this is not itself as SNA study. | 4-5 |
| Editor | Methods | Please provide additional details regarding participant consent. In the ethics statement in the Methods and online submission information, please ensure that you have specified (1) whether consent was informed and (2) what type you obtained (for instance, written or verbal, and if verbal, how it was documented and witnessed). If your study included minors, state whether you obtained consent from parents or guardians. If the need for consent was waived by the ethics committee, please include this information. | Clarification added | 8 |
| 1 | Results | I believe that the results are genuinely useful for other studies with similar objectives, as the information and conclusions provide added value for future research. | thanks :) |  |
| 2 | Results | p. 10 Line 24, punctuation | We have reviewed this line and can not see a punctuation error. But have made a modification to the text in that paragraph to aid reading. | 10 |
| 2 | Results | p. 14 Line 16-17 “and co-developed an comic” ? | Typo correct to "a comic" | 7 |
| 2 | Results | Develop justification for organizing your figure 1 in that way. Who inspired you? what are your sources? It looks like a very useful grid to understand People using drugs (PUDS) resisting help seeking: if we could use your grid to propose typical solutions to explore as interveners preparing potential therapies (topic, responses, solution hints for each of them), then we would have an intervention tool to skip dead-ends in accessing help for PUDS. Your classification of typical responses to why can't you seek for help is really needed in Harm and Risk Reduction processes. This classification is the new result in your topic as I recognize easily PUDS stories in what you observe as responses | We think it is clear that Figure 1 is a visual representation of our coding framework from the analysis. We are pleased that you think it would be a useful tool for informing intervention planning, which is something we suggest in our discussion and implications sections. | Fig 1. pg. 18 |
| 4 | Results | p. 8/Table 1: In order to understand the table, more information on the social network analysis would be helpful. What types of networks were captured, how extensive were they, and how were they elicited? The manuscript refers to the interview protocol, but this was not included in the review materials | The focus of this manuscript is the factors influencing help-seeking, rather than detailed analysis of peoples networks, which is reported elsewhere (paper in press).  However, table one shows the proportion of peoples networks who had experience of mental health, substance use or the criminal justice system, and the proportion of peoples networks who were supportive/unsupportive of help-seeking. In the data collection section we state "We asked about people in their social network, including the participant’s perceptions of each person’s attitude to help-seeking and their experience of mental health, substance use and criminal justice problems" . We have added more detail of the network members' characteristics in the supplementary file.   The reference to the protocol is provided with the link for readers interested in the specific questions asked about participants' social networks, how the software operated, and the wider study of which this is a part. | Methods, reference 39 |
| 4 | Results | p. 10, line 20: Repetition of the word “seeking.” | Thank you for this detailed feedback. This is corrected | 10 |
| 4 | Results | p. 19, line 24: Repetition of the word “first.” | Thank you for this detailed feedback. This is corrected | 19 |
| 4 | Results | More information on participants’ networks would strengthen the paper overall. Were these networks predominantly characterised by similarly disadvantaged socio-economic and cultural backgrounds? Were some networks more supportive or more destructive than others? Which participants reported more positive experiences with psychosocial help, and did this relate to network composition? Were there any gender differences in the accounts? | Thank you for your interest in the networks and their role. The focus of this manuscript is the factors influencing help-seeking, rather than detailed analysis of peoples networks. The latter is reported elsewhere (paper under review).  Table 1 shows the (high) proportions of network members who shared experiences of mental ill-health, substance use problems and criminal justice system contact. We have added a table to a supplementary file on the characteristics of people in the social networks. | Supple-mentary file |
| 4 | Results | Figure 1, p. 37: The image quality is poor, and the category system is not fully self-explanatory. Clearer category labels would be useful, for example “Desire to seek help” rather than simply “Desire.” | Thank you for pointing out the poor image quality, this is improved for the revised submission and supported by a table with definitions of each in the supplementary file. | Fig 1. Supple-mentary file |
| 5 | Results | The findings richly describe influences but less clearly explain mechanisms. Consider framing mechanisms such as: identity preservation, social surveillance, normative reinforcement, emotional resource exchange, reputational risk.  This would significantly increase theoretical sophistication. | Our paper is focused on describing the influencing factors using existing behavioural theory, and drawing from the codes and themes in our study. We found support for existing theory, albeit with need for greater attention to relational components (even though these are included they get less focus). Analysis of mechanisms from an atheoretical or alternative theoretical lens was beyond our aims, but would be a valuable addition to the literature that we have acknowledged in our conclusions. | 24 |
| 5 | Results | The categories desire, ability, and context occasionally overlap conceptually. Author should provide: clearer operational definitions, a conceptual diagram, explanation of boundaries between themes.  Readers should understand why a finding belongs to one category rather than another. | Thank you for this suggestion, we have added greater detail in introducing the themes, included clearer labelling of the framework diagram, and a table explaining the themes and subthemes in supplementary material. The complexity of human behaviour, resulted in aspects that were difficult to disentangle, as noted by the reviewer, and we have added how we addressed these in the methods, results and limitations sections. | Fig 1. Results. Supple-mentary file |
| 3 | Discussion | Third, while the findings are compelling and well supported, some of the claims in the Discussion could be more cautiously framed. In particular, references to multi-level interventions and broader system-level implications would benefit from a clearer distinction between empirically grounded findings and interpretive extrapolations. | Additional clarifications made for the need for further research on the mechanisms by which these factors influence help-seeking, and how interventions can be optimally delivered in different contexts before confident recommendations can be made. | 23 |
| 3 | Discussion | Minor revisions could further enhance clarity, particularly by reducing some repetition between the Results and Discussion sections and sharpening the articulation of the manuscript’s key contributions. | We have revised the wording of our key contributions in the discussion and conclusions sections. | 19-24 |
| 4 | Discussion | The manuscript would benefit from a more explicit reflection on the implications of recruiting mainly through services for the interpretation of the findings. | We have added further reflection on how retrospective participant accounts partly counterbalance any pro-help-seeking bias introduced through recruitment via services | 23 |
| 5 | Discussion | Expand discussion on: structural welfare context, justice system differences internationally, implications for diverse populations. | We have added acknowledgement of Scotland's free at the point of use health services and provided illustrative examples of how results may have differed in different places (e.g. more ethnically heterogenous populations, health insurance models) | 24 |
| 5 | Discussion | The reflexivity statement is commendably transparent but remains procedural. The authors should discuss: how researcher positionality shaped interpretation, interactions with marginalized participants, power dynamics during interviews.  Reflexivity should inform analysis, not only reporting compliance. | We have added depth to our reflexivity section to assure readers that our positionality was actively engaged with throughout analysis | 8 |
