# Supplementary material for: You “can’t get away from the social”: Individual, relational, and contextual influences on mental health and substance use-related help-seeking among people in contact with the criminal justice system
Source: PLoS One. 2026 Jul 30;21(7):e0354996. doi: 10.1371/journal.pone.0354996 (PMC13422872; doi:10.1371/journal.pone.0354996)
Supplement: Peer review history

# Peer review: Decision Letter 0

**DOI:** 10.1371/journal.pone.0354996.r001

**Authors:** Antonio Peña-Fernández

27 May 2026

PONE-D-26-04904

You “can’t get away from the social”: Individual, relational, and contextual influences on mental health and substance use-related help-seeking among people in contact with the criminal justice system

PLOS One

Dear Dr. Connell,

Thank you for submitting your manuscript to PLOS ONE. After careful consideration, we feel that it has merit but does not fully meet PLOS ONE’s publication criteria as it currently stands. Therefore, we invite you to submit a revised version of the manuscript that addresses the points raised during the review process.

==============================

Thank you for submitting your manuscript to our journal. Your manuscript has now been peer reviewed. We would be grateful if you could address the reviewers’ comments and submit a revised version for further consideration.

==============================

Please submit your revised manuscript by Jul 08 2026 11:59PM. If you will need more time than this to complete your revisions, please reply to this message or contact the journal office at plosone@plos.org. When you’re ready to submit your revision, log on to https://www.editorialmanager.com/pone/ and select the 'Submissions Needing Revision' folder to locate your manuscript file.

Please include the following items when submitting your revised manuscript:

- A letter that responds to each point raised by the academic editor and reviewer(s). You should upload this letter as a separate file labeled 'Response to Reviewers'.
- A marked-up copy of your manuscript that highlights changes made to the original version. You should upload this as a separate file labeled 'Revised Manuscript with Track Changes'.
- An unmarked version of your revised paper without tracked changes. You should upload this as a separate file labeled 'Manuscript'.

If you would like to make changes to your financial disclosure, please include your updated statement in your cover letter. Guidelines for resubmitting your figure files are available below the reviewer comments at the end of this letter.

If applicable, we recommend that you deposit your laboratory protocols in protocols.io to enhance the reproducibility of your results. Protocols.io assigns your protocol its own identifier (DOI) so that it can be cited independently in the future. For instructions see: https://journals.plos.org/plosone/s/submission-guidelines#loc-laboratory-protocols. Additionally, PLOS ONE offers an option for publishing peer-reviewed Lab Protocol articles, which describe protocols hosted on protocols.io. Read more information on sharing protocols at https://plos.org/protocols?utm_medium=editorial-email&utm_source=authorletters&utm_campaign=protocols.

As the corresponding author, your ORCID iD is verified in the submission system and will appear in the published article. PLOS supports the use of ORCID, and we encourage all coauthors to register for an ORCID iD and use it as well. Please encourage your coauthors to verify their ORCID iD within the submission system before final acceptance, as unverified ORCID iDs will not appear in the published article. *Only*  the individual author can complete the verification step; PLOS staff *cannot*  verify ORCID iDs on behalf of authors.

We look forward to receiving your revised manuscript.

Kind regards,

Antonio Peña-Fernández, PhD

Academic Editor

PLOS One

Journal requirements:

When submitting your revision, we need you to address these additional requirements.

1. Please ensure that your manuscript meets PLOS ONE's style requirements, including those for file naming. The PLOS ONE style templates can be found at

https://journals.plos.org/plosone/s/file?id=wjVg/PLOSOne_formatting_sample_main_body.pdf and

https://journals.plos.org/plosone/s/file?id=ba62/PLOSOne_formatting_sample_title_authors_affiliations.pdf

2. Please provide additional details regarding participant consent. In the ethics statement in the Methods and online submission information, please ensure that you have specified (1) whether consent was informed and (2) what type you obtained (for instance, written or verbal, and if verbal, how it was documented and witnessed). If your study included minors, state whether you obtained consent from parents or guardians. If the need for consent was waived by the ethics committee, please include this information.

If you are reporting a retrospective study of medical records or archived samples, please ensure that you have discussed whether all data were fully anonymized before you accessed them and/or whether the IRB or ethics committee waived the requirement for informed consent. If patients provided informed written consent to have data from their medical records used in research, please include this information.

3. Thank you for stating in your Funding Statement:

“This work was supported by the Economic and Social Research Council (https://www.ukri.org/councils/esrc/).  This grant was awarded to CC and DG (Grant Ref: ES/W012448/1).

The funders played no role in the research.”

Please provide an amended statement that declares *all* the funding or sources of support (whether external or internal to your organization) received during this study, as detailed online in our guide for authors at http://journals.plos.org/plosone/s/submit-now. Please also include the statement “There was no additional external funding received for this study.” in your updated Funding Statement.

Please include your amended Funding Statement within your cover letter. We will change the online submission form on your behalf.

4. Thank you for stating the following in the Competing Interests section:

“CC, MT and JG are employed by the University of Stirling at The Salvation Army Centre for Addiction Services and Research. The Salvation Army had no role in this study’s funding or conduct.  DG has no competing interests.”

Please confirm that this does not alter your adherence to all PLOS ONE policies on sharing data and materials, by including the following statement: "This does not alter our adherence to PLOS ONE policies on sharing data and materials.” (as detailed online in our guide for authors http://journals.plos.org/plosone/s/competing-interests). If there are restrictions on sharing of data and/or materials, please state these. Please note that we cannot proceed with consideration of your article until this information has been declared.

Please include your updated Competing Interests statement in your cover letter; we will change the online submission form on your behalf.

5. When completing the data availability statement of the submission form, you indicated that you will make your data available on acceptance. We strongly recommend all authors decide on a data sharing plan before acceptance, as the process can be lengthy and hold up publication timelines. Please note that, though access restrictions are acceptable now, your entire data will need to be made freely accessible if your manuscript is accepted for publication. This policy applies to all data except where public deposition would breach compliance with the protocol approved by your research ethics board. If you are unable to adhere to our open data policy, please kindly revise your statement to explain your reasoning and we will seek the editor's input on an exemption. Please be assured that, once you have provided your new statement, the assessment of your exemption will not hold up the peer review process.

6. We note that this data set consists of interview transcripts. Can you please confirm that all participants gave consent for interview transcript to be published?

If they DID provide consent for these transcripts to be published, please also confirm that the transcripts do not contain any potentially identifying information (or let us know if the participants consented to having their personal details published and made publicly available). We consider the following details to be identifying information:

- Names, nicknames, and initials

- Age more specific than round numbers

- GPS coordinates, physical addresses, IP addresses, email addresses

- Information in small sample sizes (e.g. 40 students from X class in X year at X university)

- Specific dates (e.g. visit dates, interview dates)

- ID numbers

Or, if the participants DID NOT provide consent for these transcripts to be published:

- Provide a de-identified version of the data or excerpts of interview responses

- Provide information regarding how these transcripts can be accessed by researchers who meet the criteria for access to confidential data, including:

a) the grounds for restriction

b) the name of the ethics committee, Institutional Review Board, or third-party organization that is imposing sharing restrictions on the data

c) a non-author, institutional point of contact that is able to field data access queries, in the interest of maintaining long-term data accessibility.

d) Any relevant data set names, URLs, DOIs, etc. that an independent researcher would need in order to request your minimal data set.

For further information on sharing data that contains sensitive participant information, please see: https://journals.plos.org/plosone/s/data-availability#loc-human-research-participant-data-and-other-sensitive-data

If there are ethical, legal, or third-party restrictions upon your dataset, you must provide all of the following details (https://journals.plos.org/plosone/s/data-availability#loc-acceptable-data-access-restrictions):

1. A complete description of the dataset

2. The nature of the restrictions upon the data (ethical, legal, or owned by a third party) and the reasoning behind them

3. The full name of the body imposing the restrictions upon your dataset (ethics committee, institution, data access committee, etc)

4. If the data are owned by a third party, confirmation of whether the authors received any special privileges in accessing the data that other researchers would not have

5. Direct, non-author contact information (preferably email) for the body imposing the restrictions upon the data, to which data access requests can be sent

7. Please include captions for your Supporting Information files at the end of your manuscript, and update any in-text citations to match accordingly. Please see our Supporting Information guidelines for more information: http://journals.plos.org/plosone/s/supporting-information.

8. If the reviewer comments include a recommendation to cite specific previously published works, please review and evaluate these publications to determine whether they are relevant and should be cited. There is no requirement to cite these works unless the editor has indicated otherwise.

9. Please review your reference list to ensure that it is complete and correct. If you have cited papers that have been retracted, please include the rationale for doing so in the manuscript text, or remove these references and replace them with relevant current references. Any changes to the reference list should be mentioned in the rebuttal letter that accompanies your revised manuscript. If you need to cite a retracted article, indicate the article’s retracted status in the References list and also include a citation and full reference for the retraction notice.

Additional Editor Comments:

Dear authors,

Thank you for submitting your manuscript to our journal. Your manuscript has now been peer reviewed. We would be grateful if you could address the reviewers’ comments and submit a revised version for further consideration.

Thank you,

Antonio

[Note: HTML markup is below. Please do not edit.]

Reviewers' comments:

Reviewer's Responses to Questions

**Comments to the Author**

1. Is the manuscript technically sound, and do the data support the conclusions?

The manuscript must describe a technically sound piece of scientific research with data that supports the conclusions. Experiments must have been conducted rigorously, with appropriate controls, replication, and sample sizes. The conclusions must be drawn appropriately based on the data presented.

Reviewer #1: Yes

Reviewer #2: Yes

Reviewer #3: Yes

**********

2. Has the statistical analysis been performed appropriately and rigorously?

Reviewer #1: Yes

Reviewer #2: Yes

Reviewer #3: N/A

**********

3. Have the authors made all data underlying the findings in their manuscript fully available?

-->The PLOS Data policy requires authors to make all data underlying the findings described in their manuscript fully available without restriction, with rare exception (please refer to the Data Availability Statement in the manuscript PDF file). The data should be provided as part of the manuscript or its supporting information, or deposited to a public repository. For example, in addition to summary statistics, the data points behind means, medians and variance measures should be available. If there are restrictions on publicly sharing data—e.g. participant privacy or use of data from a third party—those must be specified.

Reviewer #1: Yes

Reviewer #2: Yes

Reviewer #3: Yes

**********

4. Is the manuscript presented in an intelligible fashion and written in standard English?

-->PLOS ONE does not copyedit accepted manuscripts, so the language in submitted articles must be clear, correct, and unambiguous. Any typographical or grammatical errors should be corrected at revision, so please note any specific errors here.

Reviewer #1: Yes

Reviewer #2: Yes

Reviewer #3: Yes

**********

5. Review Comments to the Author

-->Please use the space provided to explain your answers to the questions above. You may also include additional comments for the author, including concerns about dual publication, research ethics, or publication ethics. (Please upload your review as an attachment if it exceeds 20,000 characters)

Reviewer #1: The research conducted addresses a current and relevant issue. The results of the study are valuable and useful for interventions related to the problem investigated. The use of qualitative research methods represents an appropriate choice for this study, and the sample size is substantial both in number and diversity, as it ensures spatial heterogeneity through the selection of different geographical areas.

I believe that within the framework analysis, the model of help-seeking behavior and access to health services should be explained more clearly and in greater detail.

I consider that the involvement of people with lived experience in the research team does not necessarily represent a strong point, as it could introduce a degree of subjectivity due to their inclusion in the research team.

Nevertheless, I believe that the results are genuinely useful for other studies with similar objectives, as the information and conclusions provide added value for future research.

Reviewer #2: p. 10 Line 24, punctuation

p. 14 Line 16-17 “and co-developed an comic” ?

Figure 1, sources or inspired from who?

Develop justification for organizing your figure 1 in that way. Who inspired you? what are your sources? It looks like a very useful grid to understand People using drugs (PUDS) resisting help seeking: if we could use your grid to propose typical solutions to explore as interveners preparing potential therapies (topic, responses, solution hints for each of them), then we would have an intervention tool to skip dead-ends in accessing help for PUDS. Your classification of typical responses to why can't you seek for help is really needed in Harm and Risk Reduction processes.

This classification is the new result in your topic as I recognize easily PUDS stories in what you observe as responses

Reviewer #3: This manuscript addresses a highly relevant and underexplored topic, offering valuable insights into help-seeking behaviours for mental health and substance use among people in contact with the criminal justice system. The study is well-positioned within the literature and presents a meaningful contribution by integrating individual, relational, and contextual dimensions through a social network-informed perspective. The qualitative approach, based on framework analysis of 50 in-depth interviews, is appropriate and provides a robust empirical basis for the proposed conceptual framework.

The manuscript is methodologically sound overall, and the conclusions are generally supported by the data. However, several aspects would benefit from further clarification to strengthen transparency and conceptual coherence.

First, the methodological section would benefit from a more explicit justification of key analytical decisions. In particular, the criteria used to determine data saturation are not fully articulated, and the rationale for the final sample size could be clarified. Additionally, while the manuscript reports the use of independent coding by multiple researchers, the process for resolving discrepancies and reaching analytic consensus is not sufficiently detailed. Providing this information would strengthen the credibility and dependability of the analysis.

Second, the manuscript refers to a broader mixed-methods social network analysis study, yet only qualitative findings are presented. While this does not detract from the value of the study, the role of the social network component within the present analysis could be more clearly articulated to avoid potential ambiguity. At times, the framing may suggest a shift toward more quantitative or structural interpretations; however, the procedures for data collection and analysis remain clearly grounded in qualitative paradigms. Clarifying this positioning would enhance conceptual clarity and coherence.

Third, while the findings are compelling and well supported, some of the claims in the Discussion could be more cautiously framed. In particular, references to multi-level interventions and broader system-level implications would benefit from a clearer distinction between empirically grounded findings and interpretive extrapolations.

Finally, the manuscript is clearly written and well organised. Minor revisions could further enhance clarity, particularly by reducing some repetition between the Results and Discussion sections and sharpening the articulation of the manuscript’s key contributions.

Overall, this is a well-conducted and valuable study with clear potential for publication. The revisions required primarily relate to clarification and transparency rather than substantive changes to the study design or analysis.

**********

6. PLOS authors have the option to publish the peer review history of their article (what does this mean?). If published, this will include your full peer review and any attached files.

-->If you choose “no”, your identity will remain anonymous but your review may still be made public.

**Do you want your identity to be public for this peer review?**  For information about this choice, including consent withdrawal, please see our Privacy Policy.

Reviewer #1: **Yes:**  Atudorei Ioana

Reviewer #2: **Yes:**  Gilbert Emond

Reviewer #3: **Yes:**  Rafael Alberto Méndez-Romero, Ph.D.

**********

[NOTE: If reviewer comments were submitted as an attachment file, they will be attached to this email and accessible via the submission site. Please log into your account, locate the manuscript record, and check for the action link "View Attachments". If this link does not appear, there are no attachment files.]

To ensure your figures meet our technical requirements, please review our figure guidelines: https://journals.plos.org/plosone/s/figures

You may also use PLOS’s free figure tool, NAAS, to help you prepare publication quality figures: https://journals.plos.org/plosone/s/figures#loc-tools-for-figure-preparation.

NAAS will assess whether your figures meet our technical requirements by comparing each figure against our figure specifications.

Attachment Submitted filename: Reviewer comments.docx

Attachment Submitted filename: Comments.docx

# Author comment: Author response to Decision Letter 1

**DOI:** 10.1371/journal.pone.0354996.r002

16 Jun 2026

Thank you for your time in providing peer review to strengthen our paper. We have uploaded a point by point response.

Attachment Submitted filename: Response to reviewers.docx

# Peer review: Decision Letter 1

**DOI:** 10.1371/journal.pone.0354996.r003

**Authors:** Antonio Peña-Fernández

16 Jul 2026

You “can’t get away from the social”: Individual, relational, and contextual influences on mental health and substance use-related help-seeking among people in contact with the criminal justice system

PONE-D-26-04904R1

Dear Dr. Connell,

We’re pleased to inform you that your manuscript has been judged scientifically suitable for publication and will be formally accepted for publication once it meets all outstanding technical requirements.

Within one week, you’ll receive an e-mail detailing the required amendments. When these have been addressed, you’ll receive a formal acceptance letter and your manuscript will be scheduled for publication.

An invoice will be generated when your article is formally accepted. Please note, if your institution has a publishing partnership with PLOS and your article meets the relevant criteria, all or part of your publication costs will be covered. Please make sure your user information is up-to-date by logging into Editorial Manager at Editorial Manager® and clicking the ‘Update My Information' link at the top of the page. For questions related to billing, please contact billing support.

If your institution or institutions have a press office, please notify them about your upcoming paper to help maximize its impact. If they’ll be preparing press materials, please inform our press team as soon as possible -- no later than 48 hours after receiving the formal acceptance. Your manuscript will remain under strict press embargo until 2 pm Eastern Time on the date of publication. For more information, please contact onepress@plos.org.

Kind regards,

Antonio Peña-Fernández, PhD

Academic Editor

PLOS One

Additional Editor Comments (optional):

Dear authors,

Thank you very much for addressing all the comments and for your patience throughout the review process. I believe that the manuscript has been strengthened considerably by the revisions, and I am pleased to recommend it for publication in our journal.

Congratulations,

Antonio

Reviewers' comments:

Reviewer's Responses to Questions

**Comments to the Author**

1. If the authors have adequately addressed your comments raised in a previous round of review and you feel that this manuscript is now acceptable for publication, you may indicate that here to bypass the “Comments to the Author” section, enter your conflict of interest statement in the “Confidential to Editor” section, and submit your "Accept" recommendation.

Reviewer #1: All comments have been addressed

Reviewer #3: All comments have been addressed

**********

2. Is the manuscript technically sound, and do the data support the conclusions?

The manuscript must describe a technically sound piece of scientific research with data that supports the conclusions. Experiments must have been conducted rigorously, with appropriate controls, replication, and sample sizes. The conclusions must be drawn appropriately based on the data presented.

Reviewer #1: Yes

Reviewer #3: Yes

**********

3. Has the statistical analysis been performed appropriately and rigorously?

Reviewer #1: Yes

Reviewer #3: Yes

**********

4. Have the authors made all data underlying the findings in their manuscript fully available?

The PLOS Data policy requires authors to make all data underlying the findings described in their manuscript fully available without restriction, with rare exception (please refer to the Data Availability Statement in the manuscript PDF file). The data should be provided as part of the manuscript or its supporting information, or deposited to a public repository. For example, in addition to summary statistics, the data points behind means, medians and variance measures should be available. If there are restrictions on publicly sharing data—e.g. participant privacy or use of data from a third party—those must be specified.

Reviewer #1: Yes

Reviewer #3: Yes

**********

5. Is the manuscript presented in an intelligible fashion and written in standard English?

PLOS ONE does not copyedit accepted manuscripts, so the language in submitted articles must be clear, correct, and unambiguous. Any typographical or grammatical errors should be corrected at revision, so please note any specific errors here.

Reviewer #1: Yes

Reviewer #3: Yes

**********

6. Review Comments to the Author

Please use the space provided to explain your answers to the questions above. You may also include additional comments for the author, including concerns about dual publication, research ethics, or publication ethics. (Please upload your review as an attachment if it exceeds 20,000 characters)

Reviewer #1: Following a review of the article, the issues raised have been addressed and clarified. I consider the article valuable and worthy of publication.

Reviewer #3: I consider that the manuscript is now suitable for publication. The authors have responded satisfactorily to the observations raised in my previous review and have introduced the necessary revisions to strengthen the manuscript. In particular, they have clarified key methodological decisions, including the rationale for stopping data collection, the considerations behind the final sample size, and the limitations associated with the sampling process. They have also provided additional detail on the analytical process, which improves the credibility, transparency, and dependability of the study.

I also appreciate the clarification regarding the social network component. The revised manuscript now makes clear that this element belongs to the broader research project and does not define the methodological nature of the present paper, which remains grounded in qualitative analysis. This adjustment helps avoid conceptual ambiguity and improves the coherence of the manuscript.

Overall, the revised version addresses the main concerns previously identified and presents a clearer, stronger, and more coherent contribution. I therefore recommend that the manuscript be accepted for publication.

**********

7. PLOS authors have the option to publish the peer review history of their article (what does this mean?). If published, this will include your full peer review and any attached files.

If you choose “no”, your identity will remain anonymous but your review may still be made public.

**Do you want your identity to be public for this peer review?**  For information about this choice, including consent withdrawal, please see our Privacy Policy.

Reviewer #1: **Yes:**  ATUDOREI Ioana Anisa

Reviewer #3: **Yes:**  Rafael Alberto Méndez-Romero

**********

# Editor report: Acceptance letter

**DOI:** 10.1371/journal.pone.0354996.r004

**Authors:** Antonio Peña-Fernández

PONE-D-26-04904R1

PLOS One

Dear Dr. Connell,

I'm pleased to inform you that your manuscript has been deemed suitable for publication in PLOS One. Congratulations! Your manuscript is now being handed over to our production team.

At this stage, our production department will prepare your paper for publication. This includes ensuring the following:

* All references, tables, and figures are properly cited

* All relevant supporting information is included in the manuscript submission,

* There are no issues that prevent the paper from being properly typeset

You will receive further instructions from the production team, including instructions on how to review your proof when it is ready. Please keep in mind that we are working through a large volume of accepted articles, so please give us a few days to review your paper and let you know the next and final steps.

Lastly, if your institution or institutions have a press office, please let them know about your upcoming paper now to help maximize its impact. If they'll be preparing press materials, please inform our press team within the next 48 hours. Your manuscript will remain under strict press embargo until 2 pm Eastern Time on the date of publication. For more information, please contact onepress@plos.org.

You will receive an invoice from PLOS for your publication fee after your manuscript has reached the completed accept phase. If you receive an email requesting payment before acceptance or for any other service, this may be a phishing scheme. Learn how to identify phishing emails and protect your accounts at https://explore.plos.org/phishing.

If we can help with anything else, please email us at customercare@plos.org.

Thank you for submitting your work to PLOS ONE and supporting open access.

Kind regards,

PLOS ONE Editorial Office Staff

on behalf of

Dr. Antonio Peña-Fernández

Academic Editor

PLOS One
